# A Genetic Screen Links the Disease-Associated Nab2 RNA-Binding Protein to the Planar Cell Polarity Pathway in *Drosophila melanogaster*

**DOI:** 10.1534/g3.120.401637

**Published:** 2020-08-17

**Authors:** Wei-Hsuan Lee, Edwin Corgiat, J. Christopher Rounds, Zenyth Shepherd, Anita H. Corbett, Kenneth H. Moberg

**Affiliations:** * Emory University School of Medicine, Department of Cell Biology, Atlanta, GA 30322; † Graduate Program in Genetics and Molecular Biology, Atlanta, GA 30322; ‡ Emory University, Department of Biology, Atlanta, GA 30322

**Keywords:** *Drosophila melanogaster*, Nab2, RNA binding protein, planar cell polarity, *GMR*, eye screen

## Abstract

Mutations in the gene encoding the ubiquitously expressed RNA-binding protein ZC3H14 result in a non-syndromic form of autosomal recessive intellectual disability in humans. Studies in *Drosophila* have defined roles for the ZC3H14 ortholog, Nab2 (aka *Drosophila* Nab2 or dNab2), in axon guidance and memory due in part to interaction with a second RNA-binding protein, the fly Fragile X homolog Fmr1, and coregulation of shared Nab2-Fmr1 target mRNAs. Despite these advances, neurodevelopmental mechanisms that underlie defective axonogenesis in *Nab2* mutants remain undefined. *Nab2* null phenotypes in the brain mushroom bodies (MBs) resemble defects caused by alleles that disrupt the planar cell polarity (PCP) pathway, which regulates planar orientation of static and motile cells via a non-canonical arm of the Wnt/Wg pathway. A kinked bristle phenotype in surviving *Nab2* mutant adults additionally suggests a defect in F-actin polymerization and bundling, a PCP-regulated processes. To test for Nab2-PCP genetic interactions, a collection of PCP mutant alleles was screened for modification of a rough-eye phenotype produced by Nab2 overexpression in the eye (*GMR>Nab2*) and, subsequently, for modification of a viability defect among *Nab2* nulls. Multiple PCP alleles dominantly modify *GMR>**Nab2* eye roughening and a subset rescue low survival and thoracic bristle kinking in *Nab2* zygotic nulls. Collectively, these genetic interactions identify the PCP pathway as a potential target of the Nab2 RNA-binding protein in developing eye and wing tissues and suggest that altered PCP signaling could contribute to neurological defects that result from loss of *Drosophila* Nab2 or its vertebrate ortholog ZC3H14.

Mutations in genes encoding RNA-binding proteins often lead to tissue-specific pathology, particularly within central nervous system (reviewed in Castello *et al.* 2013). Inactivating mutations in the human *ZC3H14* gene, which encodes a ubiquitously expressed poly(A) RNA-binding protein, are linked to a monogenic form of intellectual disability (reviewed in Fasken and Corbett 2016). Studies of murine ZC3H14 and its *Drosophila melanogaster* homolog, Nab2, indicate that Nab2/ZC3H14 share a conserved function in brain neurons. Nab2 loss alters structure of the brain mushroom body (MB) lobes and impairs memory (Kelly *et al.* 2016; Bienkowski *et al.* 2017), while *ZC3H14* loss in mice alters hippocampal morphology and decreases working memory (Rha *et al.* 2017; Collins *et al.* 2019).

*Drosophila* Nab2 protein and its homologs in other species concentrate in the nucleus, with a small fraction of the protein also detected in the cytoplasm of various cell types, including neurons (Anderson *et al.* 1993; Leung *et al.* 2009; Van Den Bogaart *et al.* 2009; Guthrie *et al.* 2011; Bienkowski *et al.* 2017; Rha *et al.* 2017). Nuclear forms of Nab2/ZC3H14 proteins in various species are implicated in control of RNA poly(A) tail length, splicing, and export into the cytoplasm (Kelly *et al.* 2014; Soucek *et al.* 2016; Rha *et al.* 2017; Morris and Corbett 2018). In *Drosophila* neurons, cytoplasmic Nab2 localizes to messenger ribonucleoprotein particles (mRNPs) that contain the Fragile-X protein homolog Fmr1 and are implicated in mRNA translational repression (Bienkowski *et al.* 2017). Murine ZC3H14 also localizes to the neuronal cytoplasm, where it co-sediments with puromycin-sensitive ribosomal fractions and localizes to axons and dendritic spines (Rha *et al.* 2017). These data indicate that Nab2/ZC3H14 likely has effects on nuclear pre-mRNA processing and cytoplasmic translation of mRNAs involved in neurodevelopment.

The majority of Nab2-regulated neuronal mRNAs are undefined, but animals lacking *Nab2* share defects in the brain mushroom bodies (MBs) with mutants in one of four genes encoding components of planar cell polarity (PCP) pathway (*dishevelled*, *prickle*, *frizzled* and *Van Gogh*) (Ng 2012; Kelly *et al.* 2016), suggesting a potential Nab2-PCP link in the brain. The PCP pathway involves two apically localized transmembrane complexes, Starry Night (also Flamingo)-Van Gogh- Prickle (Stan-Vang-Pk) and Starry night-Frizzled-Dishevelled-Diego (Stan-Fz-Dsh-Dgo), that interact across cell:cell junctions but are mutually antagonistic within cells, resulting in a polarized pattern of complex accumulation that propagates across an epithelium (reviewed in Yang and Mlodzik 2015). PCP controls the planar orientation of cells via localized effects on the F-actin cytoskeleton and contributes to a number of developmentally programmed processes, including proximal-distal hair cell orientation and axon guidance in the nervous systems of multiple species (Sato *et al.* 2006; Srahna *et al.* 2006; Inaki *et al.* 2007; Hollis and Zou 2012; Ng 2012; Vandewalle *et al.* 2013; Ackley 2014; Gombos *et al.* 2015; Avilés and Stoeckli 2016).

To investigate a Nab2-PCP link *in vivo*, a group of alleles of core and accessory PCP components was screened for modification of phenotypes produced by eye-specific *Nab2* overexpression (*GMR>Nab2*) (Pak *et al.* 2011). This candidate approach identifies multiple PCP alleles that dominantly interact with *GMR>Nab2*, and a subset of PCP alleles that also modify *Nab2* loss-of-function phenotypes. A PCP-Nab2 link is further supported by our discovery that *Nab2* mutant adults exhibit wing hair misorientation, a feature of many *Drosophila* PCP factors (reviewed in Yang and Mlodzik 2015). Collectively, these data identify the PCP pathway as a potential target of the Nab2 RNA binding protein in neurons.

## Materials and Methods

### Drosophila genetics:

Crosses were maintained in 25° humidified Shel•Lab incubators with 12hr light-dark cycles. The *Nab2* alleles *ex3* (null), *pex41* (control, *precise excision 41*), and the *EP3716* line carrying *UAS* sites upstream of the *Nab2* coding sequence, have been described previously (Pak *et al.* 2011). For autosomal alleles, five males of each candidate modifier stock (‘paternal transmission’ in Table 1; balancers correspond to those listed for each BDSC stock) were crossed to five virgin *GMR>**Nab2* females (*w-;GMR-Gal4/CyO;Nab2*^*EP3716*^*/TM6B,tub- Gal80*). For X-linked lethal alleles and the two X-linked viable alleles, five virgin females of each candidate modifier stock (‘maternal transmission’ in Table 1; balancers correspond to those listed for each BDSC stock, except for *dsh*^*6*^, *dsh*^*1*^ and *Appl*^*d*^ chromosomes, which were rebalanced over *FM7a*) were crossed to five *GMR>**Nab2* males (*w-/Y;GMR-Gal4/CyO;Nab2*^*EP3716*^*/TM6B,tub-Gal80*). Three independent crosses were set up per genotype. Lines obtained from Bloomington *Drosophila* Stock Center (BDSC): *GMR-Gal4*, *dsh*^*1*^ (Axelrod *et al.* 1998), *dsh*^*3*^ (Yanagawa *et al.* 1995), *dsh*^*6*^ (Perrimon and Mahowald 1987), *fz*^*1*^ (Park *et al.* 1994), *fz*^*JB*^ (Povelones *et al.* 2005), *vang^stmb153^* and *vang^stbm6^* (Wolff and Rubin 1998), *stan*^*fz3*^ (Rawls and Wolff 2003) *stan*^*e59*^ (Lu *et al.* 1999), *pk*^*30*^ (Green *et al.* 2000), *pk^pk-sple13^* (Gubb *et al.* 1999), *DAAM*^*A*^ (Haelterman *et al.* 2014), *DAAM*^*G1567*^ (Bellen *et al.* 2004), *Appl*^*d*^ (Torroja *et al.* 1999), *wnt4^ems23^* and *wnt4^c1^* (Cohen *et al.* 2002), *tap*^*MI10541*^ and *puc*^*MI11061*^ (Nagarkar-Jaiswal *et al.* 2015), *Rbsn5^x17^* (Morrison *et al.* 2008), *Pabp2*^*55*^ (Benoit *et al.* 2005). *fz*^*JB*^ and *pk*^*pk-sple14*^ alleles were a gift of G. Pierre-Louis (present address Valencia College, FL) and J. Axelrod.

**Table 1:** Summary of tested alleles and their effect on *GMR>Nab2* eye phenotypes. Columns include allele name, identifying stock number (Bloomington Drosophila Stock Center, BDSC), allele class and chromosome location (*e.g.*, X, 2 or 3), mode of transmission into the *GMR>Nab2* background (maternal or paternal), modifying effect (– no effect, E = enhance, S = suppress), expressivity of the effect (+ mild, ++ moderate, and +++ strong), observed penetrance, number of animals scored *(m = male, f = female), and notes on the nature of the allele and a source reference. **Note that difficulty visually scoring the *Stubble^sbd-l^* marker on the *TM1* balancer, which balances *fz^1^* in BDSC#1678, prevented a confident assessment of penetrance. Abbreviations are indicated

Allele	BDSC Stock #	Allele class (chr)	Allele transmission	Effect on *GMR>Nab2*	penetrance	# of F1 adults scored∗	Notes on rationale
$\mathrm{ds}h^{1}$	5298	hypomorph (X)	maternal	**E**^**+**^ slight size	full	43m	carries w^*a*^;K417M in DEP domain disrupts PCP (Axelrod,1998)
			maternal	**S+++** size,structure and pigment	full	48f	
$\mathrm{ds}h^{3}$	5299	amorph (X)	maternal	**S++** structure,pigment slight size	full	34f	carries w^*a*^;Δ534bp causing fs after N94 (Yanagawa,1995)
$\mathrm{ds}h^{6}$	5297	amorph (X)	maternal	**-**	full	41f	carries w^*a*^;lesion unknown (Perrimon,1987)
$fz^{1}$	1678	hypomorph (3)	maternal	**S**^**+**^ size pigment	na	balancer issue∗∗	Spontaneous, lesion unknown (Park,1994)
$fz^{\mathrm{JB}}$	na	amorph(3)	paternal	**S++** size,structure and pigment	partial	41m,46f	W355Ter in third TM domain (Povelones,2005)
$\mathrm{van}g^{\mathrm{stbm153}}$	6919	hypomorph (2)	paternal	**-**	full	23m,64f	Δ7bp causing fs after S205 (Wolff,1998)
$\mathrm{van}g^{\mathrm{stbm6}}$	6918	amorph (2)	paternal	**E**^**+**^ slight size	na	42m,46f	Δ2bp causing fs after V81 (Wolff,1998)
$\mathrm{sta}n^{\mathrm{fz3}}$	6967	hypomorph (2)	paternal	**S**^**+**^ slight,structure and pigment	na	25m,67f	Iesion unknown (Rawls,2003)
$\mathrm{sta}n^{\mathrm{e59}}$	41776	amorph (2)	paternal	**E**^**+**^ pigment loss **S**^**+**^ size	full	46m,36f	Q1838Ter (Lu,1999)
$pk^{\mathrm{30}}$	44229	Strong hypomorph (2)	paternal	**S**^**+**^ slight,structure and pigment	full	52m,68f	Δ1306bp removes *pk* and *sple* function (Green,2000)
$pk^{\mathrm{pk-sple13}}$	44230	hypomorph (2)	paternal	**-**	full	28m,40f	X-ray allele affecting *pk* and *sple* function (Gubb,1999)
$pk^{\mathrm{pk-sple14}}$	na	hypomorph (2)	paternal	**S**^**+**^ slight,size, and pigment	full	34m,41f	as above
$\mathrm{DAA}M^{A}$	52348	lethal hypomorph (X)	maternal	**S+++** size,structure and pigment	full	42f	D360V in FH3 domain (Haelterman,2014)
$\mathrm{DAA}M^{\mathrm{G1567}}$	33546	potential hypomorph (X)	maternal	**S++** size pigment	full	52f	EP insertion into 5′ region (Belen,2004)
$\mathrm{App}l^{d}$	43632	amorph (X)	maternal	**S++** structure pigment	full	34m	internal deletion (Torroja,1996)
			maternal	**S+++** structure pigment	full	29f	
$\mathrm{Wnt}4^{\mathrm{ems23}}$	6650	amorph (2)	paternal	**S+++** size,structure pigment	full	29m,37f	Q343 Ter (Cohen,2002)
$\mathrm{Wnt}4^{\mathrm{c1}}$	6651	hypomorph (2)	paternal	**S**^**+**^ slight size	na	23m,34f	in-frame deletion of E299 (Cohen,2002)
$\mathrm{ta}p^{\mathrm{MI10541}}$	55498	hypomorph	paternal	**S++** size,structure pigment	full	54m,63f	MIC gene trap 5′ UTR (Nagarkar-Jaiswal,2015)
$\mathrm{pu}c^{\mathrm{MI11061}}$	56272	hypomorph (3)	paternal	**S++** size,structure pigment	full	58m,61f	MIC gene trap intron 3 (Nagarkar-Jaiswal,2015)
$\mathrm{Rbsn}5^{\mathrm{X17}}$	39628	amorph (2)	paternal	**S**^**+**^ slight size	na	20m,27f	Q241 Ter (Morrison,2008)
$\mathrm{Pabp}2^{\mathrm{55}}$	39628	amorph (2)	paternal	**E+++** semi-lethal	full	8m,5f	Deletion of coding sequence (Benoit,2005)

### Image and data collection

Images of adult eyes were captured with a Leica MC-170 HD digital camera mounted on a Nikon SMZ800N microscope and processed with Adobe Photoshop to standardize brightness and sharpness. Eye phenotypes were categorized as ‘Enhancer (E)’, ‘Suppressor (S)’, or ‘no effect (-)’ based on visual assessment of pigmentation loss, disorganization of the retinal honeycomb structure, and quantitative measure of 2D eye size (see Table 1 and Figure 3). Eye size was determined for five eyes per condition (*i.e.*, genotype and gender) using Photoshop and standardized to the average size of five *GMR-Gal4* control eyes (males in Figure 3A and females in Figure 3B). For survival assays in Figure 4A-B, 100 non-Tubby female larvae were collected in triplicate from an intercross of *Nab2*^*ex3*^*/TM6B^Tb,Hu^* or *Nab2*^*pex41*^ (‘control’) animals, transferred to a fresh vial, and allowed to pupate. Viability was assessed by manual counting of viable, eclosed adults. Survival was calculated as the number of observed adults over the total number of larvae. For modifier alleles, *Nab2*^*ex3*^*/TM6B^Tb,Hu^* females balanced for the indicated Wg/PCP allele (*e.g.*, *dsh*^*1*^*/FM7i,Actin-GFP*, *Appl*^*d*^*/FM7,Actin-GFP*, *pk*^*pk-sple14*^*/CyO,Dfd-GFP*, or *Wnt4*^*c1*^*/CyO,Dfd-GFP*) were crossed to *Nab2*^*ex3*^*/TM6B^Tb,Hu^* males, and the viability of *modifier/+;Nab2*^*ex3*^*/Nab2^ex3^* females was determined by manual counting of viable adults as described above. To calculate the penetrance of bristle kinking in Figure 5, control (*Nab2*^*pex41*^), *Nab2*^*ex3*^, and *dsh*^*1*^*/+;;Nab2*^*ex3*^ females collected from crosses described above were examined for kinking of humeral and scutal macrochaetae characteristic of *Nab2*^*ex3*^ adults (Pak *et al.* 2011). Females with at least one kinked thoracic bristle were scored as ‘positive’ for the phenotype. Kinking penetrance was determined by dividing the percent of adult females with kinked bristles over the total number of adult females observed.

### Statistical analysis

One-way ANOVA was used to compute significance values (*p*-values indicated in Legends and denoted by asterisks*) for eye size data, survival data, and bristle kink data. PrismGraphPad Software was used to generate graphs and perform statistical tests. Sample sizes (n) and p-values are indicated in figures or legends. Modifying effects of PCP alleles on *Nab2*^*ex3*^ adult viability were quantified by as observed adult *vs.* expected adults (set as 100%) among three replicates of 100 sorted female larvae.

### Data availability

All *Drosophila* transgenic and mutant lines used in this study are freely available upon request.

## Results

### PCP alleles dominantly modify the GMR>Nab2 eye phenotype

**Figure 1:**
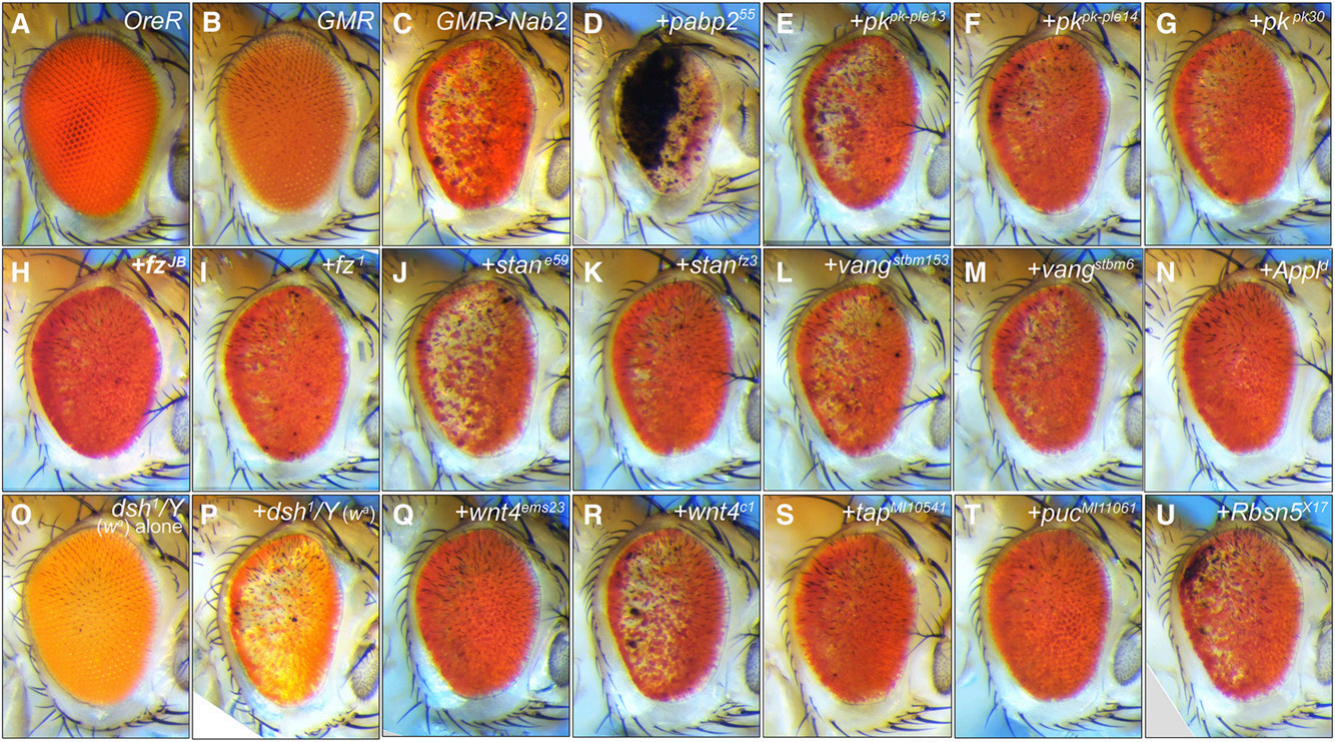
*GMR>Nab2* male eye modification by Wg/PCP alleles. Images of adult male eyes oriented posterior (left) to anterior (right): (A) *wildtype* control (*OreR*), (B) *GMR-Gal4* alone, (C) *GMR>Nab2* alone, or combined with (D) *pabp2^55^/+* (positive control as in Pak *et al*. 2011), and (E-U) the indicated Wg/PCP alleles. The (O) *w^a^,dsh^1^/Y* eye (BDSC #5298) is provided as a control for the apricot eye color and PCP-defective genetic background of the (P) *w^a^,dsh^1^/Y*, *GMR>Nab2* genotype. Images to scale.

**Figure 2:**
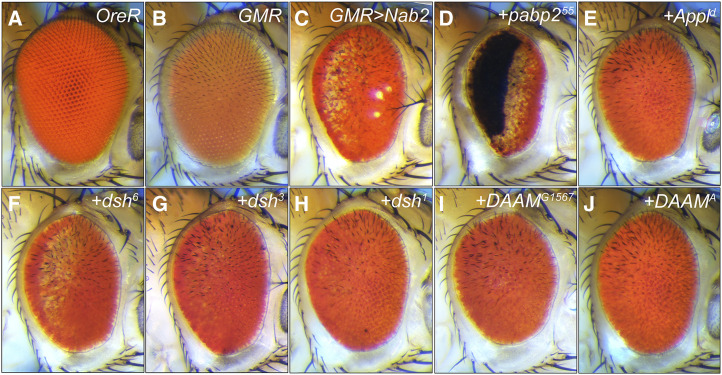
*GMR>Nab2* female eye modification by Wg/PCP alleles. Images of adult female eyes oriented posterior (left) to anterior (right): (A) *wildtype* control (*OreR*), (B) *GMR-Gal4* alone, (C) GMR>Nab2 alone, or combined with (D) *pabp2^55^/+* (positive control as in Pak *et al*. 2011), and (E-J) the indicated X-linked Wg/PCP alleles. Images to scale.

As described in prior work (Pak *et al.* 2011), overexpression of *Nab2* in developing eye tissue with a *GMR-Gal4* transgene (chromosome 2) and an EP-type transposon located in the first non-coding exon of *Nab2* (*GMR-Gal4*, *Nab2*^*EP3716*^; hereafter ‘*GMR>Nab2*’), leads to adult eyes that are rough, reduced in size, and lack red pigmentation in posterior domains relative to wildtype *Oregon-R* (*OreR*) or ‘*GMR* only’ control eyes. These phenotypes are more severe in males (Figures 1A-C) than females (Figures 2A-C) and provide a useful background to screen for alleles that dominantly interact with *Nab2*. This approach has proven effective in identifying factors that interact functionally or physically with Nab2, including the nuclear poly(A) binding protein Pabp2 (included as a positive control in Figures 1D and 2D) (Pak *et al.* 2011) and the disease-associated RNA-binding protein Fmr1 (see Bienkowski *et al.* 2017).

**Figure 3:**
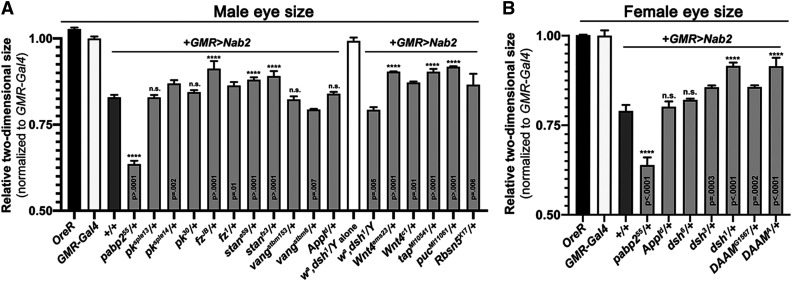
Effect of Wg/PCP alleles on two-dimensional size of *GMR>Nab2* eyes. Quantitation of 2D eye size in adult (A) males or (B) females carrying the indicated alleles. Pixel number was determined for five eyes per test genotype using Photoshop and normalized to five *GMR-Gal4* control eyes. Asterisks denote modification with P<0.0001. Other p values are noted. ’n.s.‘ = not significant. Errors bars represent SEM.

To use the *GMR>Nab2* transgenic system to assess genetic interactions between Nab2 and PCP factors, a group of twenty (20) alleles corresponding to core and accessory PCP factors (Table 1) was crossed into the *GMR>Nab2* background and scored for modification of eye size, roughening, and pigmentation in the F1 progeny. Fourteen autosomal alleles were screened in *GMR>Nab2* males (Figure 1E-U), and four X-linked lethal alleles of the Wg/PCP factors *disheveled* (*dsh*^*3*^, *dsh*^*6*^) and *Dishevelled Associated Activator of Morphogenesis* (*DAAM*^*G1567*^, *DAAM*^*A*^) were tested as heterozygotes in *GMR>**Nab2* females (Figure 2E-J). Two viable X-linked mutant alleles, *dsh*^*1*^ and *Amyloid precursor protein-like^d^* (*Appl*^*d*^), were tested both as hemizygotes in males (Figure 1N,P) and heterozygotes in females (Figure 2E,H). As summarized in Table 1, seventeen of the alleles modified, to varying degrees, one of the three *GMR>Nab2* eye phenotypes tracked in this study: loss of pigmentation, disorganized ommatidial structure, and reduced eye size. Modification of the first two phenotypes was assessed by visual inspection, while the effect on male and female eye size was determined by measuring two-dimensional (2D) eye size in fixed images (Figures 3A, B). Nine of the alleles produced moderate (S++) or strong suppression (S+++) of one or more of the three scored *GMR>Nab2* phenotypes: *dsh*^*3*^, *frizzled*^*JB*^ (*fz*^*JB*^), *DAAM*^*A*^, *DAAM*^*G1567*^, *Appl*^*d*^, *Wnt oncogene analog-4^ems23^* (*Wnt4*^*ems23*^), *target of Poxn*^*MI10541*^ (*tap*^*MI10541*^), and *puckered*^*MI11061*^ (*puc*^*MI11601*^). One allele, *vang^stbm6^*, mildly enhanced (E+) *GMR>**Nab2* and six alleles mildly suppressed (S+) one or more of the *GMR>Nab2* phenotypes: *fz*^*1*^, *starry night*^*fz3*^ (*stan*^*fz3*^), *pk*^*30*^, *pk*^*pk-sple14*^, *Wnt4*^*c1*^, and *Rbsn5^X17^*. One allele, *stan*^*e59*^, mildly enhanced *GMR>**Nab2* pigment loss, but also mildly suppressed the reduced eye size. Finally, the *dsh*^*1*^ allele suppressed eye size, pigment loss and roughness in *dsh*^*1*^*/+* heterozygous females, but had a mild enhancing effect on *GMR>**Nab2* in hemizygous males (*i.e.*, *dsh*^*1*^*/Y*), although this phenotype was more difficult to score based on the apricot eye color of the *w^a^* allele (Morgan *et al.* 1925) carried on the *dsh*^*1*^ chromosome (see Figure 1C *vs*. O,P). Overall, of the twenty tested Wg/PCP alleles, only three, *dsh*^*6*^, *vang^stmb153^* and *pk*^*pk-sple13*^, had no effect on *GMR>Nab2* eye phenotypes.

A review of the alleles with modifying effects is consistent with a link between Nab2 and the Wg/PCP pathway. Two of three *dsh* alleles tested (*dsh*^*1*^ and *dsh*^*3*^) suppressed the female *GMR>**Nab2* phenotype as heterozygotes. The *dsh*^*3*^ amorph carries an internal deletion that causes a frameshift after codon 94 (Yanagawa *et al.* 1995), and the *dsh*^*1*^ hypomorph selectively blocks PCP signaling due to an amino acid substitution in the DEP domain (Dishevelled, E gl-10, P leckstrin) (Axelrod *et al.* 1998; Boutros *et al.* 1998; Penton *et al.* 2002). *GMR>**Nab2* male eyes are mildly enhanced by *dsh*^*1*^*/Y* hemizygosity, perhaps due to a requirement for residual wildtype Dsh function to modify Nab2 phenotypes or to a sex-specific interaction between *dsh*^*1*^ and *GMR>**Nab2*. Both *fz* alleles tested act as dominant suppressors, and the *fz*^*JB*^ amoprh is a stronger suppressor than the *fz*^*1*^ hypomorph. *stan*^*fz3*^ is also a suppressor, although a second allele, *stan*^*e59*^, has mild enhancing and suppressing effects on *GMR>**Nab2* phenotype. Among *pk* alleles tested, *pk*^*30*^ (also *Df(2R)pk*) and *pk*^*pk-sple14*^ mildly suppress, while a third allele, *pk*^*pk-sple13*^, has no obvious effect on *GMR>**Nab2* eyes.

Alleles of PCP accessory factors also strongly modify the *GMR>**Nab2* phenotype. Two alleles of the X-linked Dsh-interactor *DAAM* act as strong dominant suppressors of *GMR>**Nab2* eye phenotypes; one of these suppressors, *DAAM*^*A*^, contains a D360V substitution in the formin-homology 3 (FH3) domain (Haelterman *et al.* 2014) that is predicted to disrupt interactions with Rac GTPases during axonal outgrowth (Gombos *et al.* 2015). The suppressor allele *Appl*^*d*^ (β-Amyloid p rotein p recursor-like) is an amorph that deletes the central coding region of a transmembrane protein that acts as a PCP-accessory factor in neurons and has established roles in retinal axon pathfinding and synapse formation (Luo *et al.* 1992; Ashley *et al.* 2005; Mora *et al.* 2013; Soldano *et al.* 2013). The amorphic allele *Wnt4*^*ems23*^, which encodes a Fz ligand with roles in canonical and non-canonical Wg/Wnt signaling (Cohen *et al.* 2002), is a strong suppressor, while the weak hypomorph *Wnt4*^*c1*^ has a much weaker effect. Viable *P*-element insertions in the *puc* (*puc*^*MI11060*^) and *tap* (*tap*^*MI10541*^) loci, which respectively encode a component of the PCP-regulated JNK pathway (Boutros *et al.* 1998; Martin-Blanco *et al.* 1998) and a regulator of Dsh levels in brain mushroom body (MB) neurons (Yuan *et al.* 2016), also act as *GMR>**Nab2* suppressors. Finally, a mutant allele of the endocytic factor *Rabenosyn-5* (*Rbsn5*), which regulates polarized distribution of PCP proteins in wing cells (Mottola *et al.* 2010) has a mild suppressive effect on the size of *GMR>**Nab2* eyes. In sum, these data reveal a series of genetic interactions which are consistent with a model in which reducing PCP activity partially mitigates the effect of Nab2 overexpression in the developing eye.

### PCP alleles dominantly modify Nab2 loss of function phenotypes

The genetic interactions between multiple PCP alleles and the *GMR>**Nab2* overexpression transgene prompted analysis of PCP allele interactions with the *Nab2* loss-of-function (LOF) allele *Nab2*^*ex3*^, a recessive amorph that causes defects in survival, lifespan, locomotion, thoracic bristle morphology, and neurodevelopment (Pak *et al.* 2011; Kelly *et al.* 2016). Four different PCP alleles – three suppressor alleles, *dsh*^*1*^, *Appl*^*d*^, *pk*^*pk-sple-14*^, and one weak modifier allele, *Wnt4*^*C1*^ - were tested for dominant effects on two readily scored *Nab2*^*ex3*^ null phenotypes: reduced survival to eclosion (<5% in past studies *e.g.*, Pak *et al.* 2011), and thoracic bristle kinking. The *Nab2*^*ex3*^ survival defect was chosen because it is rescued by neuron-specific expression of either fly *Nab2* or human *ZC3H14* (Kelly *et al.* 2016), suggesting that it is an indicator of a conserved requirement for Nab2 in neurons. The *Nab2*^*ex3*^ thoracic bristle defect was chosen because it suggests a link to the PCP-regulated processes of F-actin assembly and bundling (reviewed in Adler and Wallingford 2017).

**Figure 4:**
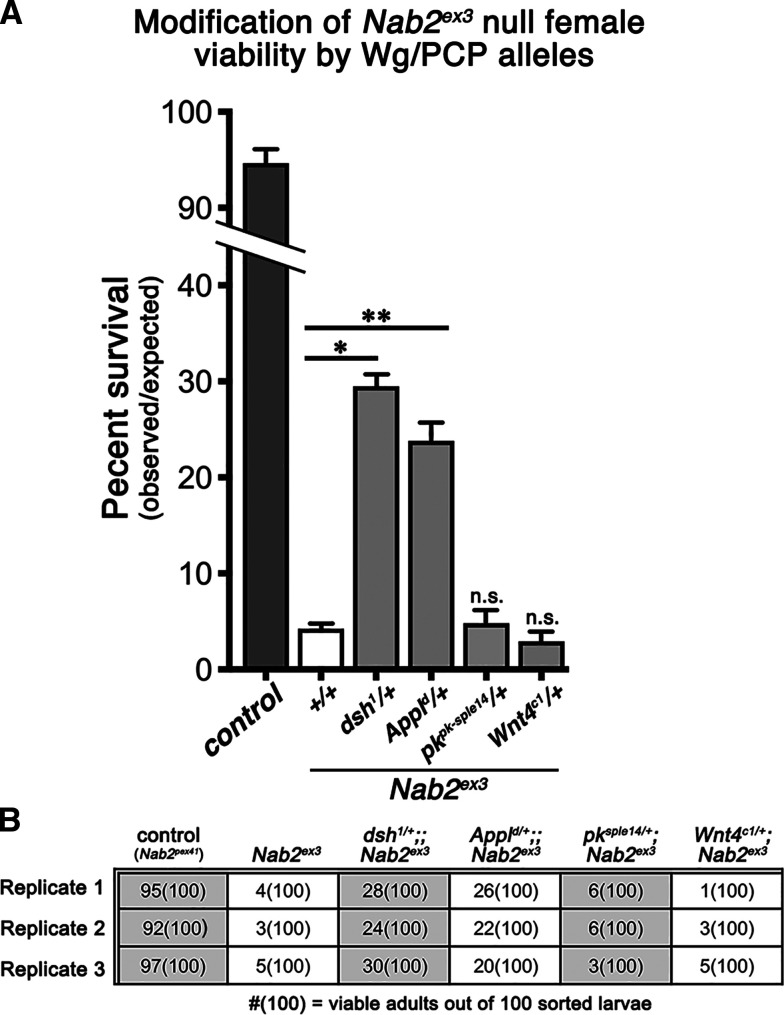
Effect of four Wg/PCP alleles on survival of *Nab2* null females. (A) Quantification of female eclosion rates among *wildtype* controls (dark gray fill; *Nab2*^*pex41*^), *Nab2*^*ex3*^ homozygotes (white fill), or *Nab2*^*ex3*^ homozygotes that are also heterozygous for *dsh*^*1*^, *Appl*^*d*^, *pk*^*pk-sple-14*^ or *Wnt4*^*c1*^ (gray fill). Data are presented as the percentage of viable females (observed) *vs.* the total number of pupae tracked (expected) from three biological replicates of 100 sorted female larvae shown in (B) (also see Materials and Methods). Statistical significance is indicated (∗p=0.0002; ∗∗p=0.0005; n.s. = not significant). Error bars represent SEM.

**Figure 5:**
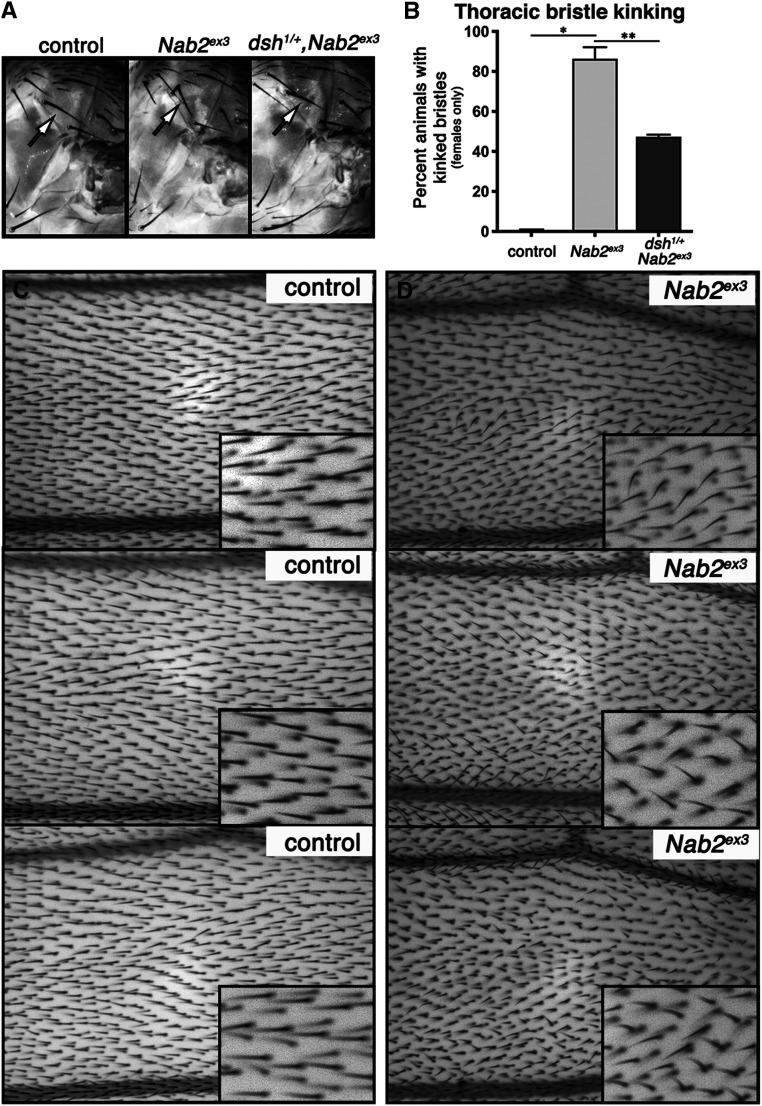
*Nab2*^*ex3*^ effects on bristle morphology and wing hair orientation. (A) Examples of humeral bristle morphology (arrows) in a *wildtype* control (*Nab2*^*pex41*^), *Nab2*^*ex3*^ homozygote and *dsh*^*1/+*^*;;Nab2*^*ex3*^ adult female. (B) Frequency of bristle kinking in *wildtype* controls (*Nab2*^*pex41*^), *Nab2*^*ex3*^ homozygotes (gray fill) or *dsh*^*1/+*^*;;Nab2*^*ex3*^ (dark gray fill) adult females. Data are presented as the percentage of viable females with at least one kinked humeral or scutal bristle *vs.* the total number of eclosed females counted from three biological replicates of 100 sorted female larvae (see Materials and Methods). Statistical significance is indicated (∗p=0.0003; ∗∗p=0.02). (C-D) Images of wing hairs in the L3-L4 region from three representative examples of control (*Nab2pex*^*41*^) or *Nab2*^*ex3*^ adult female wings orientated proximal to distal (right to left). Insets (lower right) show magnified views from each panel.

To analyze adult viability, the percent of pupae eclosing into viable adults was calculated among 100 non-Tubby female larvae collected from an intercross of *Nab2*^*ex3*^*/TM6B^Tb,Hu^* adults. Consistent with our prior work (*e.g.*, Pak *et al.* 2011), approximately 3–5% of *Nab2*^*ex3*^ zygotic null females eclose as viable adults, while *Nab2*^*pex41*^ control females (aka *Nab2*^*wt*^; a precise excision of the *EP3716* *P*-element that was used to generate *Nab2*^*ex3*^) display essentially full viability (∼94% eclosion) (Figure 4A-B). The X-linked *dsh*^*1*^ and *Appl*^*d*^ alleles respectively rescue *Nab2*^*ex3*^ female survival to ∼30% and ∼25% when inherited through *FM7a*-balanced heterozygous mothers. Heterozygosity for either the *pk*^*pk-sple14*^ or *Wnt4*^*c1*^ alleles has no significant effect on *Nab2*^*ex3*^ female survival. These genetic data demonstrate that two PCP alleles that modify *GMR>Nab2*, *dsh*^*1*^ and *Appl*^*d*^, also dominantly rescue the partial lethality of *Nab2*^*ex3*^ females. This viability rescue in *dsh*^*1*^^/+^*;;Nab2*^*ex3*^ surviving adult females is also associated with rescue of thoracic bristle kinking compared to *Nab2*^*ex3*^ null females (∼45% *vs.* ∼90%; calculated as fraction of individuals showing at least one kinked humeral or scutal bristle) (Figure 5A-B).

The ability of PCP alleles to modify phenotypes associated with Nab2 gain (*GMR>Nab2*) and loss (*Nab2*^*ex3*^) prompted an assessment of whether *Nab2* loss is sufficient to produce PCP-like defects in sensitive tissues *e.g.*, altering proximal-distal wing hair orientation (Yang and Mlodzik 2015). To test this hypothesis, hairs in a fixed region between L3-L4 veins and distal to the posterior cross vein (PCV) were imaged in control (*Nab2*^*pex41*^) and *Nab2*^*ex3*^ adult female wings (Figures 5C-D). Three examples of adult wing hair morphology in the L3-L4 region are provided for each genotype, together with corresponding magnified insets. Wing hairs in control wings are arrayed in a uniform right-to-left orientation that matches the proximal-to-distal axis of the wing (Figure 5C); by contrast, hairs in *Nab2*^*ex3*^ wings exhibit orientation defects (Figure 5D), including misrotated hairs and occasional evidence of coordinated misrotation among adjacent hair cells (*e.g.*, Figure 5D, inset in top panel). These PCP-like effects in *Nab2* null females provide evidence of a role for Nab2 in orientating adult wing hairs that is consistent with dominant genetic interactions detected between PCP mutant alleles and a *Nab2* LOF allele and a *UAS-Nab2* transgene.

## Discussion

Here we have used three phenotypes caused by altered dosage of the *Drosophila* poly(A) RNA-binding protein Nab2, (1) eye roughness caused by overexpression of *Nab2* (*GMR>Nab2*), (2) a neuronal requirement for *Nab2* in adult survival and (3) thoracic bristle kinking in *Nab2* null animals, to screen for genetic interactions between *Nab2* and genes encoding components of the Wg/planar cell polarity (PCP) pathway. The *Nab2*-PCP interactions detected by this approach are consistent with a role for Nab2 in restraining PCP signaling in *Drosophila* tissues, perhaps by inhibiting expression of a PCP component. Given that PCP signaling and Nab2/ZC3H14 are each linked to axon guidance (Sato *et al.* 2006; Srahna *et al.* 2006; Inaki *et al.* 2007; Pak *et al.* 2011; Hollis and Zou 2012; Ng 2012; Vandewalle *et al.* 2013; Ackley 2014; Kelly *et al.* 2014; Gombos *et al.* 2015; Avilés and Stoeckli 2016; Kelly *et al.* 2016; Bienkowski *et al.* 2017; Rha *et al.* 2017; Collins *et al.* 2019), these data argue that neurodevelopmental defects in flies, mice, and humans lacking Nab2/ZC3H14 may arise in part due to altered PCP signaling.

Alleles that impair Wg/PCP signaling, and in some cases specifically perturb PCP (*e.g.*, *dsh*^*1*^, *Appl*^*d*^, *DAAM*^*A*^), are able to suppress phenotypes caused by either *Nab2* overexpression (*GMR>Nab2*) or loss (*Nab2*^*ex3*^). This pattern differs from other *Nab2* modifier alleles that have inverse effects in *Nab2* gain *vs.* loss backgrounds *e.g.*, *dfmr1^∆50^* (as in Bienkowski *et al.* 2017), and could be explained if Nab2 modulates levels of a PCP factor(s). Since overexpression and loss-of-function of some PCP factors, like Frizzled, produce similar disruptions to polarity (reviewed in Yang and Mlodzik 2015), gain and loss of Nab2 might also be expected to have similar effects on PCP that are suppressed by Wg/PCP alleles. Imbalanced PCP signaling resulting from Nab2 overexpression or loss would then be restored by reducing the genetic dose of Wg/PCP factors. The suppressive effects of Wg/PCP amorphs like *dsh*^*3*^ and *fz*^*JB*^ do not distinguish whether Nab2 could affect one or both arms of the Wg/PCP pathway. The suppressive effect of the *dsh*^*1*^ allele, which specifically impairs PCP signaling (Axelrod *et al.* 1998), provides evidence of a Nab2 link to the PCP pathway. If this link is strong enough, then *Nab2* null flies might thus be expected to display defects in hallmark PCP-regulated processes, *e.g.*, wing hair polarization and ommatidial rotation. Consistent with this hypothesis, we document moderate hair misorientation defects in *Nab2* null adult wings, suggesting that Nab2 may regulate PCP activity in this tissue. Nab2 is expressed ubiquitously but required in neurons for viability (Kelly *et al.* 2014); thus, the rescue of *Nab2* null viability by *dsh*^*1*^ also implies that a Nab2-PCP link may also occur in neurons. This idea is further supported by the genetic interaction between *Nab2*^*ex3*^ and the *Appl*^*d*^ allele, which inactivates a neuron-specific PCP component (Soldano *et al.* 2013) and by *GMR>Nab2* modification by an allele of *tap*, a regulator of Dsh expression in neurons (Yuan *et al.* 2016). Interestingly, *Nab2* and PCP alleles individually alter the trajectories of mushroom body axons that project from Kenyon cells (Ng 2012; Kelly *et al.* 2016), which provides a cellular context for future study of a Nab2-PCP interaction.

Nab2 may modify PCP-regulated developmental processes indirectly through post- transcriptional control of proteins (or a protein) that is not core PCP components but regulates a common process (*e.g.*, via F-actin bundling). Alternatively, Nab2 may directly regulate post-transcriptional expression of a core PCP component. In this regard, it is notable that levels of the Vang family member Vangl2 are elevated in the hippocampal proteome of *Zc3h14* knockout mice (Rha *et al.* 2017), and that depletion of ZC3H14 from cultured N2A cells leads to intron retention in the *PSD95* mRNA (Morris and Corbett 2018), which encodes a postsynaptic guanylate kinase required for activity-dependent synaptic plasticity (reviewed in Xu 2011). Intriguingly, the fly PSD95 homolog Discs Large-1 (Dlg1) controls canonical Wg signaling in wing tissue by stabilizing Dsh protein (Liu *et al.* 2016). In light of these observations in mammalian systems, the corresponding *Drosophila* mRNAs *vang* and *dlg1* represent candidate Nab2 targets that may contribute to Nab2-PCP genetic interactions documented in this study.

In summary, we have carried out a candidate-based genetic screen that has identified a series of dominant genetic interactions between Wg/PCP alleles and both a *Nab2* overexpression transgene and null allele. Based on data cited above, these genetic data support a molecular model in which Nab2 may regulate expression of a PCP protein or proteins in different cell types, including neurons and wing hair cells. This hypothesis is significant given that very few Nab2-target mRNAs are known and the Nab2 human ortholog ZC3H14 is lost in an inherited form of recessive intellectual disability (Pak *et al.* 2011). Thus, the work presented here is a key first step in exploring conserved functional and molecular links between Nab2/ZC3H14 and PCP activity that may contribute to a conserved requirement for Nab2/ZC3H14 in neurodevelopment.
